# The Effects of *In Ovo* Injection of Synbiotics on the Early Growth Performance and Intestinal Health of Chicks

**DOI:** 10.3389/fvets.2021.658301

**Published:** 2021-06-07

**Authors:** Ao-yi Duan, An-qi Ju, Ying-nan Zhang, Yan-jun Qin, Li-gang Xue, Xin Ma, Wei-min Luan, Shu-bao Yang

**Affiliations:** ^1^Center for Reproductive Medicine, Jilin Medical University, Jilin, China; ^2^College of Animal Science and Technology, Jilin Agricultural University, Changchun, China; ^3^School of Public Health, Jilin Medical University, Jilin, China; ^4^College of Life Science, Changchun Sci-Tech University, Changchun, China

**Keywords:** synbiotics, *in ovo* injection, growth performance, immune function, intestinal health

## Abstract

In this study, the effects of synbiotic inclusion at the intra-amniotic stage in layer chicks were evaluated with different parameters, such as performance, immunological function, intestinal development, and cecal microflora content. A total of 1,200 eggs with fertile embryos were allocated into four treatment groups. For every treatment, five replicates were used, and 60 eggs were included in each replicate. The following four treatment groups were established: the non-injected group, 0.9% physiological saline injection (saline) group, 1 × 10^6^ CFU/egg *Lactobacillus plantarum* injection (probiotic) group, and 1 × 10^6^ CFU/egg *L. plantarum* + 2 mg/egg Astragalus polysaccharide injection (synbiotic) group. *In ovo* injection was carried out at 18.5 days of incubation. The results showed that *in ovo* injection of probiotics or synbiotics did not affect the hatching or growth performance of the chicks but significantly increased their feed intake (FI), body weight (BW), and the feed conversion ratio (FCR). Additionally, *in ovo* injection of synbiotics enhanced the levels of serum interleukin-2 (IL-2), interferon-γ (IFN-γ), and secretory immunoglobulin A (SIgA) in intestinal lavage fluid and the histomorphological development of the small intestine. Our results also indicated that intra-amniotic synbiotic injection significantly increased *Lactobacillus* and *Bifidobacterium* colonization while decreasing the relative abundance of *Escherichia coli* in the chicken cecum (*P* < 0.05). In summary, *in ovo* injection of synbiotics had positive impacts on the performance, immunological function, gut development, and microbiota of growing chicks.

## Introduction

At the initial stages of hatching, the immune system of the chick is immature and inefficient. When exposed to the external environment, the pollutants and pathogenic bacteria in the environment may enter the chicks' bodies, causing diseases, and affecting their growth and development (1). During the prehatch period, providing appropriate nutrition may help minimize the incidences of diseases by increasing the availability of certain nutrients (2). *In ovo* injection has been reported to be an effective way to deliver nutrients into the amniotic cavity to compensate for the energy deficiencies that occur during the hatching process (3). Considering the susceptibility of newly hatched chicks to various pathogens, administering *Lactobacillus* before hatching may be used as a strategy to enhance the immune-reactivity of hatchlings while reducing their susceptibility to pathogens (4). The first barrier against infection in poultry is the intestinal immune system. Thus, beneficial bacteria quickly colonize the intestines of hatchlings to help them establish a good balance of gut microflora, which can competitively inhibit pathogenic microorganisms in the intestine and promote disease resistance (5). Early colonization of beneficial bacteria in the intestinal tract not only can prevent intestinal disorders related to pathogenic bacteria but can also promote the maturity and integrity of the intestinal tract (6). Therefore, any improvement in early intestinal maturation and digestive ability shows a positive impact on the growth and production performance of chicks (7). Advances in intra-amniotic administration techniques have enabled the incorporation of several nutrients or active compounds in late embryos, these substances are then swallowed, digested, and absorbed before hatching occurs (8). Previous studies have shown that, in addition to vaccines, vitamins, peptides, and minerals can be injected into the amniotic cavity near hatching (9), while more recent studies have confirmed that probiotics, prebiotics, and synbiotics can also be injected into fertilized eggs without causing harmful effects (10, 11). Different studies have reported beneficial influences of probiotics on chick growth performance, intestinal microflora, and immune function (12, 13).

Among the probiotics administered to chicks, the genus *Lactobacillus* has received much attention due to its biological activities and intestinal health benefits. The species of *Lactobacillus* are considered autochthonous residents in the gastrointestinal tract of chicks. They may promote host intestinal health and immune function in different ways, such as by strengthening the epithelial barrier, competitively rejecting pathogenic microorganisms, producing antimicrobial substances, and interacting with immune cells by stimulating pattern recognition receptors (14). Many studies have suggested that different *Lactobacillus* strains can regulate many aspects of the immune response, including the expression of cytokines and chemokines and the number of T lymphocytes and systemic antibody-mediated responses (15, 16). Astragalus polysaccharide (APS) is the main component extracted from Astragalus and has multiple biological activities, including immunomodulation, antiviral, antitumor, and antioxidation activities (17). Previous studies have indicated that the oral intake of APS or its use as a feed additive can promote intestinal development and modulate the intestinal mucosal immunity of chicks (18, 19). APS and *Lactobacillus* may interact to affect immunity and intestinal microbiota because APS is an effective prebiotic additive that can increase the abundances of beneficial bacteria (*Lactobacillus* and *Bifidobacteria*) (20). Moreover, *Lactobacilli* are able to reduce intestinal pH and ferment indigestible prebiotics and further enhance the biological activity of prebiotics (21).

There have been few recent studies on *in ovo* injection of laying hens, and most of these are on broilers. No studies have explored the combined effects of the intra-amniotic application of *L. plantarum* and APS. The early development and maturation of the intestinal tract can guarantee the growth of laying chicks and the health and production performance of layer hens. Early regulation of nutrition, at the embryonic stage, not only can improve the growth and development of chicks but can also affect the growth and health of the animal throughout the whole life cycle (22). In this study, layer chicks were used as animal models to study the effects of injecting synbiotics (a combination of *L. plantarum* and APS) *in ovo* on early growth performance and intestinal health to provide a theoretical basis for the application of the *in ovo* injection of synbiotics in layer chicks.

## Materials and Methods

### Experimental Design and Egg Incubation

A total of 1,400 fertilized Hy-Line Sonia chicken eggs were obtained from a commercial hatchery (Runcheng Breeding Factory, Changchun, China). The dry powder of *Lactobacilli* (*L. plantarum*, total viable count ≥5 × 10^11^ CFU/g) and Astragalus polysaccharide (APS) was provided by Shenyang Fengmei Biotechnology Co., Ltd. (Liaoning, China). After the eggshells were disinfected with 1 ml L^−1^ of bromo-geramine solution, the eggs were set inside the incubator trays. The eggs were incubated under standard conditions in a Hongtai incubator (HTC-8, Hongtai incubation equipment factory, China). At 18.5 days of incubation, all the eggs were candled. The unfertilized or nonviable eggs were removed from the incubator, and 1,200 eggs with fertile embryos were allocated into four treatment groups. For every treatment, five replicates were established, and 60 eggs were included in each replicate. The following four treatment groups were stablished: the non-injected group, 0.9% physiological saline injection (saline) group, 1 × 10^6^ CFU/egg *L. plantarum* injection (probiotic) group, and 1 × 10^6^ CFU/egg *L. plantarum* + 2 mg/egg APS (synbiotic) group. Synbiotic dose optimization was determined as previously described (23) with minor modifications based on our preliminary experiments. On 18.5 days of incubation, the eggs were separately injected *in ovo* with the solutions described above. Before injection, all eggs were sterilized by spraying with 75% ethanol. Additionally, the instruments were autoclaved before use. Then, a 1-mm^2^ hole was punctured aseptically with a drill on top of each egg to deliver the prepared solutions. Next, 0.2 ml of the solution was injected into each individual amniotic cavity using a 28.5-gauge needle. After the injection, the holes in the eggs were immediately sealed with paraffin, and then, the eggs were returned to the incubator. The *in ovo* injection procedure was generally completed within 30 min.

### Animal Housing

The initial egg weight (EW) was recorded before incubation. As previously described by Tian et al. (24), after hatching, the numbers of live hatched and unhatched chicks were counted to calculate the hatchability (%) of fertilized eggs per replicate. The hatchling chick weight (HW) of each chick was determined, and each chick was sexed. Eighty female layer hatchling chicks from each of the four treatment groups were randomly assigned to five replicate cages with 16 chicks in each cage. Three chicks from each treatment replicate group were sacrificed and weighed for the determination of chick body weight (BW) (g); feed intake (FI) per replicate was recorded from day 1 to 21 post-hatch, and the feed conversion ratio (FCR) was calculated by dividing the body weight gain (BWG) with the FI. The birds were reared for 3 weeks with feed and water provided *ad libitum*. A layer pullet mash starter diet was formulated to meet the National Research Council (NRC) requirements (NRC, 1994) in metabolizable energy (ME) levels (2800 kcal/kg) and crude protein (CP) level (17%) and provided for the entire 3 weeks duration of the study. All the experimental protocols were approved by the Animal Care and Welfare Committee of Jilin Agricultural University.

### Sample Collection

On days 1, 7, 14, and 21 posthatch, one chick (five chicks per treatment) with a BW close to the average BW of the replicate was selected and weighed after feed deprivation for 8 h. Then, blood was collected from the cervical vein immediately after sacrifice. The thymus, spleen, and bursa of Fabricius from each chicken were prepared and weighed. The small intestine was maintained on ice and carefully separated into the duodenum, jejunum, and ileum. Intestinal lavage fluid was prepared from the jejunum and ileum samples. For further analysis, serum and intestinal lavage fluid were stored at −20°C. For morphological analysis, the small intestine was rinsed with 0.75% cold aseptic saline and fixed with 4% buffered formaldehyde (pH 7.4) for 24 h. In addition, the cecum was collected to analyze the gut microbiota, and cecal contents were frozen in liquid nitrogen and stored at −80°C until DNA extraction.

### The Measurement of Immune Organs

We weighed the immune organs, such as the thymus, spleen, and bursa of Fabricius, on days 1, 7, 14, and 21. The data are presented as a percentage of the weight of the specific immune organ of the total BW. The index was calculated as immune organ weight divided by BW and multiplied by 100%.

### The Concentration of Serum Cytokines and Intestinal Lavage Fluid SIgA

Blood samples were collected and centrifuged at 2,000 rpm for 15 min at 4°C and then stored at −80°C until analysis. Five-centimeter sections of the jejunum and ileum were removed, infused with 0.5 mL of PBS (pH 7.4, supplemented with 0.1% BSA and aprotinin) and washed three times. The washed samples were collected and centrifuged at 2,000 rpm for 15 min at 4°C. The supernatants were collected and stored at −20°C until further analysis. Commercial ELISA kits (Lengton Bioscience Co., Ltd., Shanghai, China) were used to determine the concentrations of cytokines, namely, serum IL-2 and IFN-γ, along with levels of SIgA in the intestinal lavage fluid.

### The Observation of Intestinal Histology

The duodenum, jejunum, and ileum were isolated, fixed in 4% buffered formaldehyde, and routinely embedded in paraffin. To measure the morphology, 5-μm cross-sections were prepared and stained using common hematoxylin–eosin (HE) staining as described by Sobolewska et al. (25). Intestinal histology of the cross-sections was visually examined using a light microscope (Olympus BX41, Olympus Optical Co. Ltd., Tokyo, Japan) and analyzed using Image-Pro Plus 6.0 software (Media Cybernetics, Bethesda, USA). VH was measured from the top of the villus to the crypt mouth, and CD was defined as the depth of the invagination between adjacent crypt mouths. At least five well-oriented and intact villus-crypt units were examined from the intestinal cross-section of each section per chicken.

### Gut Microbiota Analysis

The cecum was removed, and ~20–30 mg of cecal content was weighed. Total cecal DNA was extracted using DNA Δ Simple Total DNA kit-DP431 (Tiangen, Beijing, China) following the manufacture's specification. The sequences of primers that were used to target genes, including *Bifidobacterium* spp. (26), *Lactobacillus* spp. (27), and *E. coli* (28) as well as universal bacteria (29) used as a housekeeping control, are presented in Table 1. As previously described by Sun et al. (30), each 20 μl of quantitative PCR (qPCR) reaction mixture contained 1 μg of complementary DNA (cDNA) and specific primers at a final concentration of 1 μM. The specificity of the primers was verified by analysis of melting curves. During the PCR, samples were subjected to an initial denaturation phase at 95°C for 5 min, followed by 35 cycles of denaturation at 95°C for 15 s, and annealing and extension at 58°C for 30 s. The PCR fragments were purified, recovered, and connected to a pMD18T carrier. Accordingly, the plasmid OD_260_ was used to calculate an efficiency parameter. Efficiency = 6.02 × 10^23^ (efficiency/mol) DNA quantity/DNA length (bp)/660 (g/mol/bp). The relative abundance of the bacteria in the cecal content was calculated using the formula: relative abundance (%) = (efficiency universal)^Ct universal^/(efficiency target)^Ct target^.

**Table 1:** The primer sequences used for qRT-PCR.

**Gene name**	**Primer**	**Sequence (5′ → 3′)**	**Tm (°C)**
Universal bacteria	Forward	TGATATTGCTGCGCTCGTTG	58
	Reverse	CTTTCTGGCCCATACCAACC	
*Bifidobacterium* spp.	Forward	GCGTGCTTAACACATGCAAGTC	58
	Reverse	CACCCGTTTCCAGGAGCTATT	
*Lactobacillus* spp.	Forward	AGCAGTAGGGAATCTTCCA	58
	Reverse	CACCGCTACACATGGAG	
*E. coli*	Forward	CATGCCGCGTGTATGAAGAA	58
	Reverse	CGGGTAACGTCAATGAAA	

### Statistical Analysis

Statistical analysis was performed by one-way analysis of variance (ANOVA) using SPSS v20.0 (SPSS Inc., Chicago, IL, USA) and GraphPad PRISM v7.0 (GraphPad Software Inc, La Jolla, CA). The differences among the treatments were examined using Duncan's multiple range test. The arithmetic mean was calculated along with the standard error (SE) of the mean. The differences were considered statistically significant if *P* < 0.05.

## Results

### Hatching and Growth Performance

The changes in hatching and growth performance are presented in Table 2. The initial egg weights (EWs) ranged from 60.54 to 60.68 g, with no significant differences observed between the initial weight of the eggs and the weights of 18 embryonic eggs among all the groups (*P* > 0.05). Administration of synbiotics to the embryonated eggs on day 18.5 of incubation did not lead to any effect on the hatching weight (HW). Additionally, there was no significant difference in hatchability among the groups (*P* > 0.05). Table 2 shows the changes in FI, BWG, and FCR for 21 days posthatch. In contrast to the period between 8 and 21 days, no significant differences in FI, BWG, or FCR were observed among the treatments before day 7 post-hatch (*P* > 0.05). The FI and BWG of the probiotic and synbiotic groups were higher (*P* < 0.05) than those of the non-injected and saline groups 8–14 and 15–21 days post-hatch. The synbiotic group showed a significantly increased FCR compared to the other groups (*P* < 0.05). Pronounced changes were observed between the probiotic and synbiotic groups for the BWG and FCR (*P* < 0.05).

**Table 2:** Effects of *in ovo* injection of synbiotics on hatching and growth performance of chicks.

**Item**	**Groups**				***P***
	**Non-injected**	**Saline**	**Probiotic**	**Synbiotic**	
EW (g)	60.68 ± 0.06	60.57 ± 0.05	60.65 ± 0.07	60.54 ± 0.10	0.778
HW (g)	41.99 ± 0.19	42.25 ± 0.11	42.38 ± 0.05	42.35 ± 0.02	0.469
Hatchability (%)	89.33 ± 0.12	91.50 ± 0.17	90.23 ± 0.06	90.67 ± 0.15	0.587
**1–7 days**					
FI (g)	301.71 ± 0.57	302.11 ± 1.10	302.34 ± 1.37	302.78 ± 0.64	0.415
BWG (g)	30.88 ± 0.63	31.56 ± 0.23	31.85 ± 0.36	32.53 ± 0.78	0.399
FCR (g g^−1^)	9.77 ± 0.10	9.57 ± 0.10	9.49 ± 0.15	9.31 ± 0.55	0.132
**8–14 days**					
FI (g)	611.54 ± 3.83^b^	612.44 ± 2.90^b^	634.44 ± 1.03^a^	635.78 ± 2.53^a^	0.045
BWG (g)	31.57 ± 1.57^c^	31.49 ± 0.52^c^	35.51 ± 1.69^b^	39.04 ± 2.46^a^	0.038
FCR (g g^−1^)	5.16 ± 0.25^c^	5.14 ± 0.59^c^	5.59 ± 0.74^b^	6.28 ± 1.09^a^	0.040
**15–21 days**					
FI (g)	923.36 ± 9.67^b^	925.22 ± 6.05^b^	940.85 ± 5.71^a^	944.63 ± 3.55^a^	0.019
BWG (g)	41.31 ± 2.49^c^	42.04 ± 1.27^c^	48.89 ± 0.11^b^	55.60 ± 2.86^a^	0.024
FCR (g g^−1^)	4.47 ± 0.71^c^	4.54 ± 0.49^c^	5.16 ± 0.46^b^	5.89 ± 0.26^a^	0.032

*EW, initial egg weight; HW, hatching weight; FI, feed intake; BWG, body weight gain; FCR, the ratio of BWG to FI*.

*In the same row, values with the no letter or the same letter superscripts mean no significant difference (P > 0.05), while with different small letter superscripts mean significant difference (P < 0.05). The results are represented as the mean ± SE (n = 5)*.

### The Immune Organ Index

The changes in the immune organ index are presented in Table 3. Throughout the study period, the chicks grew well without incidence of disease or death. There were no pronounced changes in the immune organ index on day 1(*P* > 0.05). On days 14 and 21, the thymus and spleen organ indices in the probiotic and synbiotic groups were significantly higher than those of the two control groups (*P* < 0.05), while on days 7 and 21, the thymus index was significantly higher in the synbiotic group than in the probiotic group (*P* < 0.05). On day 14, both the probiotic and synbiotic groups showed a significant increase in the bursa of Fabricius index compared to the two control groups (*P* < 0.05).

**Table 3:** Effects of *in ovo* injection of synbiotics on the immune organ index of chicks.

**Items**	**Day**	**Groups**				***P***
		**Non-injected**	**Saline**	**Probiotic**	**Synbiotic**	
Thymus index	1 day	2.64 ± 0.21	2.98 ± 0.20	2.82 ± 0.43	2.81 ± 0.15	0.309
	7 days	2.39 ± 0.21^b^	2.39 ± 0.28^b^	2.77 ± 0.28^a^	2.98 ± 0.35^a^	0.020
	14 days	3.37 ± 0.18^b^	3.38 ± 0.18^b^	3.82 ± 0.11^a^	3.85 ± 0.13^a^	0.017
	21 days	3.73 ± 0.19^c^	3.72 ± 0.18^c^	4.07 ± 0.22^b^	4.39 ± 0.15^a^	0.015
Spleen index	1 day	0.38 ± 0.08	0.36 ± 0.13	0.32 ± 0.11	0.39 ± 0.06	0.638
	7 days	0.53 ± 0.13	0.58 ± 0.09	0.54 ± 0.06	0.57 ± 0.10	0.862
	14 days	0.59 ± 0.05^b^	0.61 ± 0.07^b^	0.65 ± 0.09^ab^	0.76 ± 0.06^a^	0.046
	21 days	0.55 ± 0.28^b^	0.63 ± 0.08^b^	0.74 ± 0.11^a^	0.79 ± 0.21^a^	0.011
Bursa of fabricius index	1 day	0.96 ± 0.17	0.83 ± 0.26	0.88 ± 0.18	1.03 ± 0.17	0.427
	7 days	0.97 ± 0.19	0.93 ± 0.23	1.44 ± 0.56	1.22 ± 0.36	0.138
	14 days	2.11 ± 0.09^b^	2.2 ± 0.25^b^	2.69 ± 0.25^a^	2.76 ± 0.34^a^	0.001
	21 days	3.71 ± 0.63	3.78 ± 0.53	3.63 ± 0.49	3.60 ± 0.41	0.947

*In the same row, values with the no letter or the same letter superscripts mean no significant difference (P > 0.05), while with different small letter superscripts mean significant difference (P < 0.05). The results are represented as the mean ± SE (n = 5)*.

### The Levels of Serum Cytokines and Intestinal Lavage Fluid SIgA

**Figure 1:**
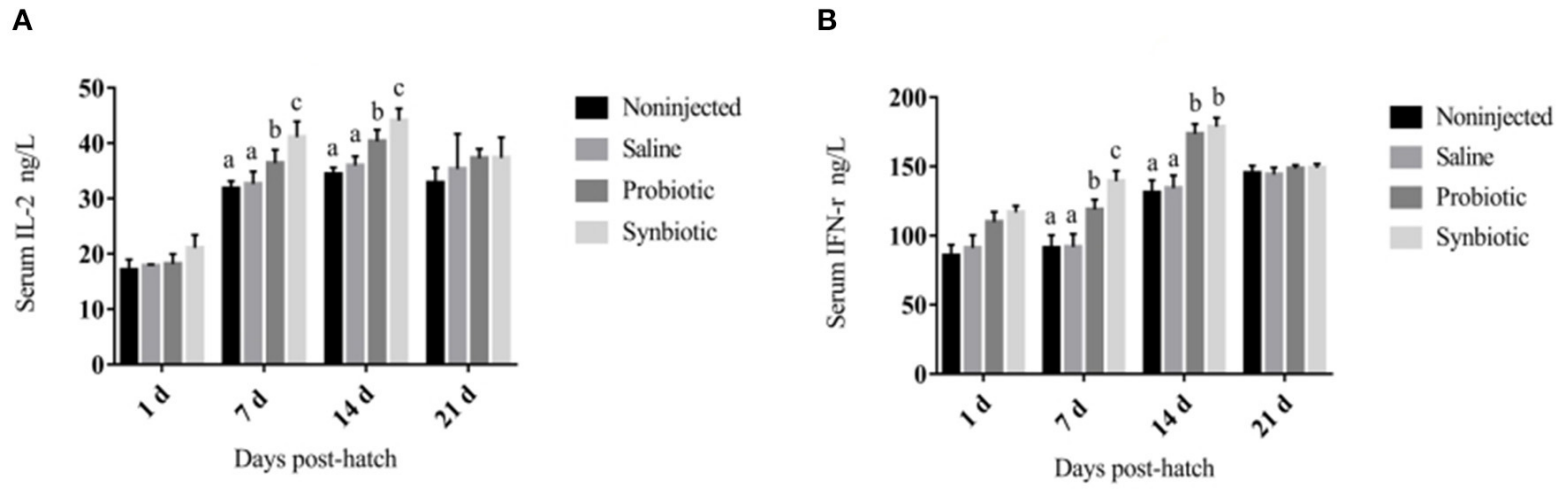
Serum IL-2 and IFN-γ concentrations in the chicks (ng/L). **(A)** IL-2; **(B)** IFN-γ. Different letters on the column charts indicate a significant difference among the treatment groups (*P* < 0.05), no letter or the same letter on the column charts mean no significant difference (*P* > 0.05). The results are represented as the mean ± SE (*n* = 5).

**Figure 2:**
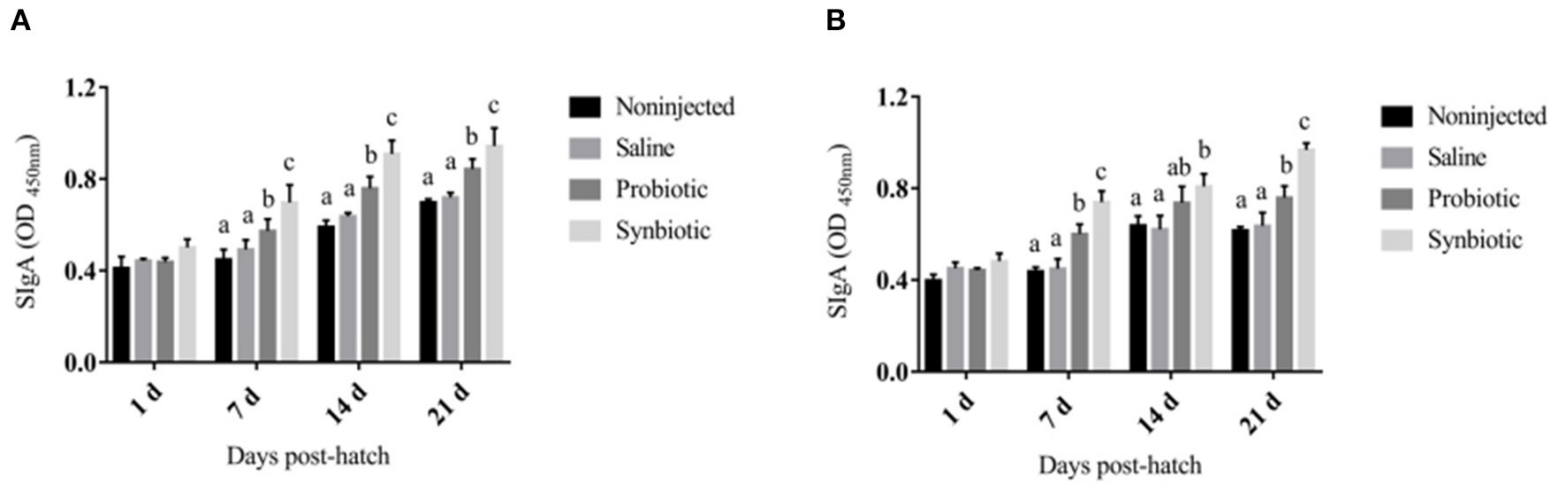
Intestinal lavage fluid SIgA level in the chicks (OD_450nm_). **(A)** Duodenum lavage fluid; **(B)** Jejunum lavage fluid. Different letters on the column charts indicate a significant difference among the treatment groups (*P* < 0.05), no letter or the same letter on the column charts mean no significant difference (*P* > 0.05). The results are represented as the mean ± SE (*n* = 5).

The changes in the levels of serum cytokines are shown in Figure 1. On days 7 and 14, a significant increase was observed in the concentrations of serum IL-2 (Figure 1A) and IFN-γ (Figure 1B) in the probiotic and synbiotic groups (*P* < 0.05) compared to the non-injected and saline groups. The changes observed in the levels of SIgA in the intestinal lavage fluid are shown in Figure 2. Compared to the two control groups, a significant increase was observed in the level of SIgA in the duodenal lavage fluid 1 day after *in ovo* injection in the probiotic and synbiotic groups (*P* < 0.05, Figure 2A). The level of SIgA in the synbiotic group was found to be the highest among the four groups and was also significantly higher than that of the probiotic group (*P* < 0.05, Figure 2A). Compared to the non-injected and saline groups, the level of SIgA in the jejunal lavage fluid in the synbiotic group showed a significant increase on days 7, 14, and 21 (*P* < 0.05, Figure 2B).

### Intestinal Histological Analyses

The data analysis of histomorphological changes in the duodenum, jejunum, and ileum is shown in Table 4; compared to the non-injected and saline groups, a significant increase was observed in the VH of the duodenum layers between days 14 and 21. Similarly, a significant increase was observed on days 14 and 21 in the VH of the jejunum and the VH of the ileum in the probiotic and synbiotic groups after the *in ovo* injection (*P* < 0.05). The VH/CD ratios of the duodenum, jejunum, and ileum were found to be increased (*P* < 0.05) in the synbiotic group compared to those of the two control groups. The CD in the intestine of the probiotic and synbiotic groups was found to be lower (*P* < 0.05) than that of the non-injected and saline groups. However, upon receiving a synbiotic injection, the CD in the eggs on day 21 showed no significant changes in the duodenum (*P* = 0.051) and jejunum (*P* = 0.097).

**Table 4:** Effects of synbiotics *in ovo* injection on the intestinal morphology of chicks.

**Items**	**Day**	**Groups**					***P***
			**Non-injected**	**Saline**	**Probiotic**	**Synbiotic**	
Duodenum	VH (μm)	14 days	831.12 ± 5.03^c^	835.86 ± 13.42^c^	855.47 ± 8.23^b^	876.17 ± 8.47^a^	0.001
		21 days	939.17 ± 5.25^c^	943.47 ± 4.68^c^	982.25 ± 7.05^b^	995.37 ± 6.41^a^	0.000
	CD (μm)	14 days	131.25 ± 9.20^a^	131.28 ± 5.16^a^	114.52 ± 11.01^b^	112.70 ± 7.18^b^	0.041
		21 days	134.67 ± 3.23	131.69 ± 6.25	120.46 ± 4.49	119.72 ± 10.27	0.051
	VH/CD	14 days	6.35 ± 0.45^b^	6.37 ± 0.32^b^	7.51 ± 0.66^a^	7.79 ± 0.45^a^	0.012
		21 days	6.98 ± 0.20^b^	7.17 ± 0.30^**b**^	8.16 ± 0.34^a^	8.36 ± 0.76^a^	0.013
Jejunum	VH (μm)	14 days	662.06 ± 10.96^c^	667.29 ± 9.20^bc^	683.55 ± 12.53^ab^	690.39 ± 10.43^a^	0.039
		21 days	706.90 ± 11.51	711.69 ± 18.04	717.55 ± 15.71	722.54 ± 9.99	0.581
	CD (μm)	14 days	118.14 ± 5.42^a^	113.04 ± 6.59^a^	99.22 ± 3.94^b^	94.25 ± 8.18^b^	0.005
		21 days	116.53 ± 3.03	112.84 ± 6.50	108.32 ± 5.22	104.61 ± 5.55	0.097
	VH/CD	14 days	5.61 ± 0.17^b^	5.91 ± 0.34^b^	6.90 ± 0.39^a^	7.36 ± 0.59^a^	0.002
		21 days	6.07 ± 0.12^c^	6.32 ± 0.31^b^	6.64 ± 0.20^b^	6.92 ± 0.30^a^	0.039
Ileum	VH (μm)	14 days	500.03 ± 14.97	509.06 ± 10.27	518.73 ± 8.41	511.05 ± 13.16	0.358
		21 days	556.29 ± 6.30^b^	559.79 ± 8.90^b^	578.22 ± 8.03^a^	584.00 ± 9.12^a^	0.008
	CD (μm)	14 days	65.97 ± 1.13	67.11 ± 3.08	65.70 ± 1.61	66.19 ± 4.42	0.936
		21 days	113.50 ± 6.28^a^	109.14 ± 8.99^a^	95.27 ± 4.89^b^	87.44 ± 2.84^b^	0.003
	VH/CD	14 days	7.58 ± 0.10	7.60 ± 0.51	7.90 ± 0.12	7.74 ± 0.33	0.602
		21 days	4.91 ± 0.22^c^	5.15 ± 0.39^c^	6.08 ± 0.40^b^	6.68 ± 0.11^a^	0.000

*VH, villus height; CD, crypt depth; VH/CD, the ratio of villus height to crypt depth*.

*In the same row, values with the no letter or the same letter superscripts mean no significant difference (P > 0.05), while with different small letter superscripts mean significant difference (P < 0.05). The results are represented as the mean ± SE (n = 5)*.

**Figure 3:**
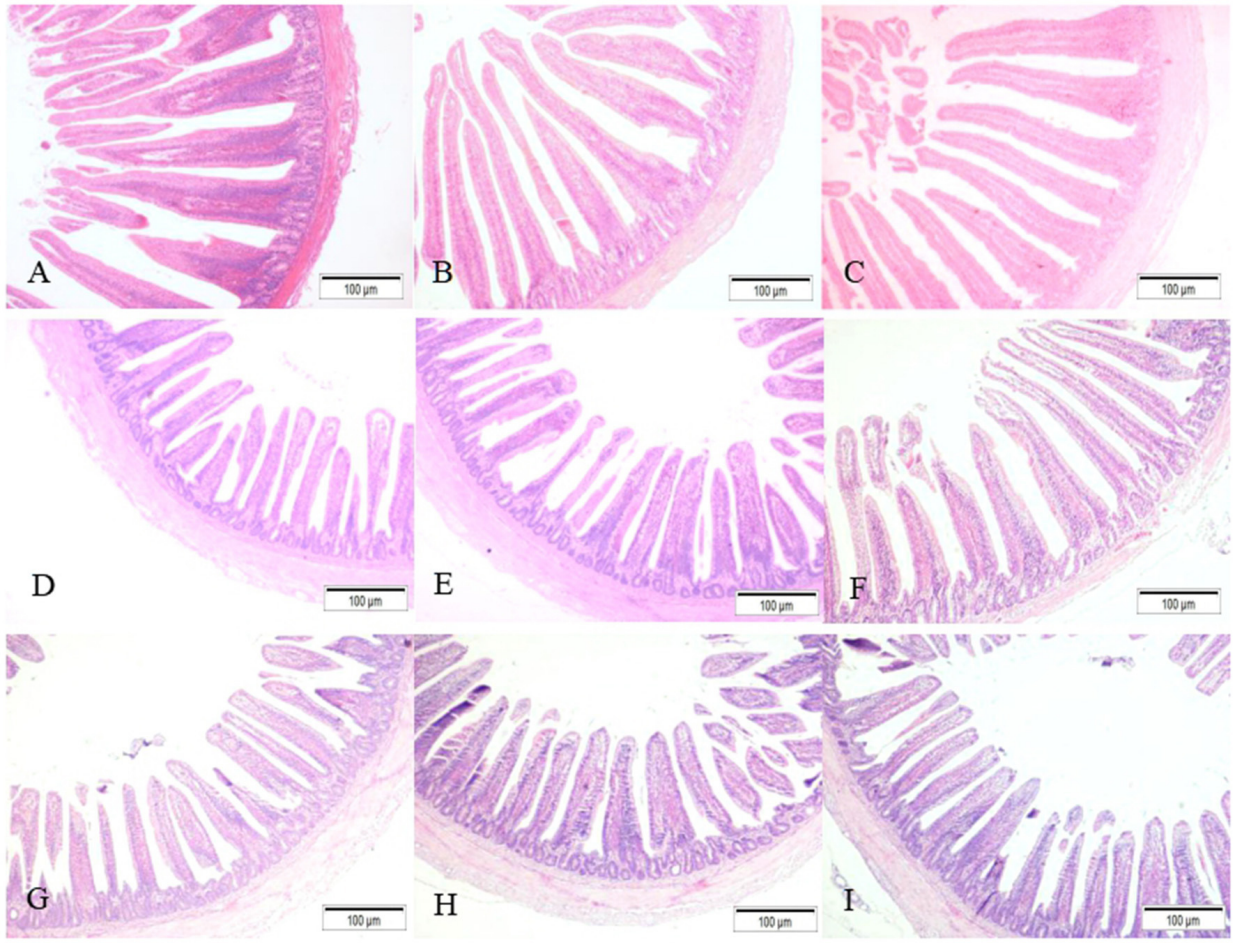
The development of the intestinal morphological structure as observed in chicks from different groups (HE staining, 100×). **(A–C)** The duodenum in the non-injected, probiotic, and synbiotic groups on day 14; **(D–F)** the jejunum in the non-injected, probiotic, and synbiotic groups on day 14; **(G–I)** the ileum in the non-injected, probiotic, and synbiotic groups on day 21.

The intestinal morphological changes are shown in Figure 3, compared to the noninjected group (Figure 3A), the duodenal villi in the probiotic and synbiotic groups (Figures 3B,C) were in a closer and more orderly array on day 14. The jejunal villus in the synbiotic group (Figure 3F) grew higher than those in the non-injected and probiotic group (Figures 3D,E) on day 14. The villi of ileum from the probiotic and synbiotic groups (Figures 3H,I) were longer and wider than those in the non-injected group (Figure 3G) on day 21.

### Gut Microbiota

**Figure 4:**
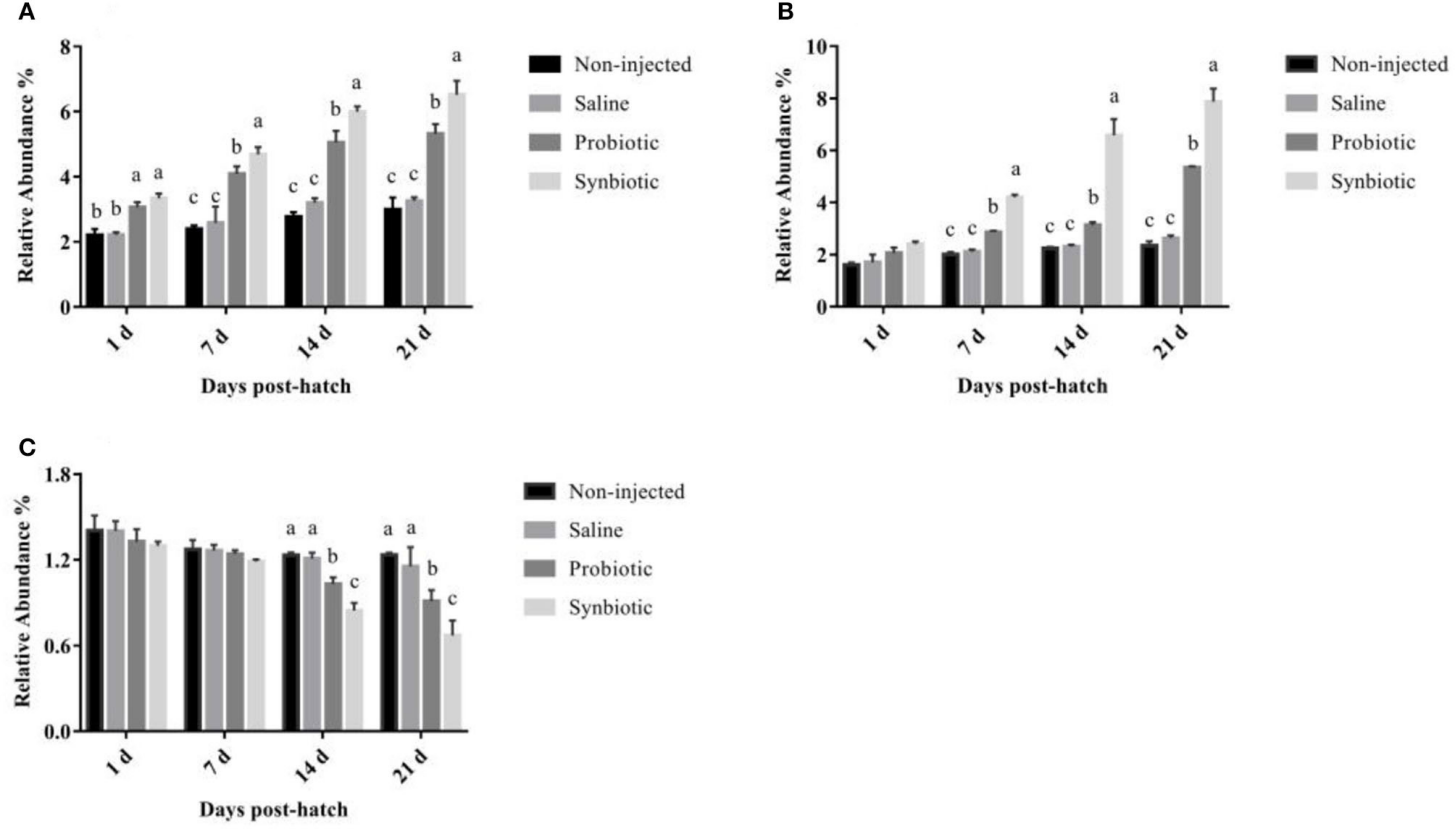
The relative abundance of **(A)** *Lactobacillus* spp., **(B)** *Bifidobacterium* spp., and **(C)** *E. coli* in the chyme from the cecum of chicks. Different letters on the column charts indicate a significant difference among the treatment groups (*P* < 0.05); no letter or the same letter on the column charts mean no significant difference (*P* > 0.05). The results are represented as the mean ± SE (*n* = 5).

The effects of *in ovo* injection of synbiotics on the relative abundance of *Lactobacillus* spp., *Bifidobacterium* spp., and *E. coli* are shown in Figure 4. Compared to the non-injected and saline groups, the relative abundance of *Lactobacillus* spp. differed significantly in the probiotic and synbiotic groups (*P* < 0.05, Figure 4A) after *in ovo* injection. In the cecum of the synbiotic group, the relative abundance of *Bifidobacterium* spp. was quickly increased on day 7 (*P* < 0.05, Figure 4B), but the relative abundance of *E. coli* decreased rapidly on day 14 compared to that of the non-injected and saline-treated chicks (*P* < 0.05, Figure 4C).

## Discussion

Earlier studies have shown that synbiotics can promote the production performance of chicks by improving their digestive ability, regulating their intestinal flora, increasing the immune system response, and ultimately improving their overall health (31). The immune system of chicks matures during embryonic development. The normal development of the immune system is inseparable from the regulation of cytokines released by immune system cells. The intestinal cavity, mucosal surface, and cecum of chicks are filled with different microorganisms. The balance of these gut microbes is important to maintain the growth and development of modern antibiotic-free chicks (6). Additionally, the early colonization of healthy microorganisms in the intestine of hatchlings has been shown to have a significant effect on their performance and their future intestinal health (32, 33).

Our results showed that *in ovo* injection of synbiotics did not affect the hatchability or HW of the chicks. These findings were consistent with those of previous studies, which reported that 100 ng of growth hormone in the albumen and amnion injection with 10^4^ CFU lactic acid bacteria (FloraMax®-B11) on day 18 of incubation increased the BW of chicks but did not affect their hatchability (34). Our results indicated that, upon *in ovo* injection, the combination of *L. plantarum* and APS facilitated the establishment of healthy microflora in the intestine without negatively affecting hatchability. As indicated in previous studies, *in ovo* injection of probiotics, prebiotics, or synbiotics can have favorable impacts on the hatching of chicks (9). However, further research is needed to develop commercial standards and observe broad effects of *in ovo* injection of these materials in practice. Wang et al. showed that the basal diet supplemented with probiotic (*Diutina rugosa* SD-17) significantly increased BW from week 3 to 6 and the FCR in weeks 1 and 2 of layer chickens (35). In this study, *in ovo* injection of synbiotics significantly increased the FI and BWG between day 7 and 21 posthatch. These results indicate that synbiotics significantly promoted the early growth performance of chicks.

The increased immune organs index observed in our study in response to the *in ovo* administration of synbiotics can be generally attributed to the better health status of the chickens. Specifically, this effect may be due to the stimulation of a protective immune response that enhances resistance to microbial pathogens. Furthermore, previous studies have also shown the stimulation of the immune response can be produced in conjunction with the increase of BW and immune organs index in chicks following the dietary administration of synbiotics (36, 37). In this study, the relative weights of the spleen, thymus, and bursa of Fabricius were assessed as direct index of immune stimulation. Our results showed that the administration of synbiotics improved the indices of all lymphoid organs with a pronounced effect on both the thymus and spleen.

The ability of humoral and cellular immunity to play crucial roles in the defense mechanism against infectious diseases is reflected by the antibody levels. It has been reported that probiotics can enhance intestinal cell-mediated mucosal immunity by stimulating heterophil bactericidal mechanisms and altering the expression of anti-inflammatory cytokines of the intestinal mucosal probiotic organisms (38, 39). IL-2 and IFN-γ are both important cytokines that play fundamental roles in stimulating the proliferation of B and T lymphocytes by inhibiting the production of proinflammatory modulators (40). In the present study, on days 7 and 14, a significant increase in serum IL-2 and IFN-γ was observed in the probiotic and synbiotic groups (*P* < 0.05) compared to the non-injected and saline groups. The SIgA produced by activated B cells is the most important aspect of the mucosal adaptive immune system, which forms a protective layer on the intestinal mucosal surface. This mechanism also requires cytokines with immunomodulatory activity to guard against the incursion of harmful pathogens (41). Our results showed that the synbiotics could enhance the serum cytokine and intestinal lavage fluid SIgA levels. We inferred that synbiotics may have also stimulated the humoral immune system to produce more antibodies, which could adhere to the intestinal mucous membrane surface to reduce villus damage.

Intestinal morphology is an important determinant of digestion and absorption of intestinal functions, and information on the relationship between synbiotics and intestinal morphology is already available. Morphological changes in the small intestine, such as increasing VH and the VH/CD ratio, can improve the performance of chicks by enhancing the absorptive surface area, which is important for the application of alternative growth stimulators (5). A higher value of VH/CD also indicates a higher rate of maturity and functional capacity of enterocytes (42). To this end, Cheled-Shoval et al. (7) showed that compared with the physiological saline injection group, *in ovo* injection with mannan oligosaccharide (MOS) significantly increased the VH, CD, and goblet cell numbers of chicks. Sobolewska et al. (25) confirmed the positive effect of *in ovo* injections on the villi of the duodenum, jejunum, and ileum 1 day after prebiotic and synbiotic administration to chicks. Therefore, *in ovo* injection of different kinds of prebiotics and synbiotics generally has led to obvious improvements in intestinal morphology. The integrity of intestinal mucosal VH and CD is the most important indicator of intestinal mucosal function. A longer VH indicates an increased nutritive absorption area and enhanced secretion and immune function of the intestinal barrier. Our study showed that the administration of synbiotics consisting of *L. plantarum* and APS contributed to maximized VH and VH/CD ratios. This effect could have promoted the faster development of the digestive tract and subsequent immune function during the hatching period and cause a significant increase in FI and FCR, ultimately resulting in a significant improvement in growth performance.

*Lactobacillus* are commensal bacteria that can activate intestinal immune function and enhance disease resistance along with producing short-chain volatile fatty acids (SCFAs) and bacteriocins, both of which may inhibit gut colonization by pathogens. Moreover, some *Lactococcus* strains have anti-inflammatory activity (43) that can inhibit certain harmful enteric bacteria. PCR analysis of template DNA isolated from the feces of chickens on days 1, 21, and 42 indicated the survivability of *Lactococcus lactis* in chicken guts after *in ovo* injection (44). Facilitating early colonization of the digestive tract by these bacteria via *in ovo* injection of synbiotics may prevent infection and improve the development and maturation of the chicken immune system.

Intestinal microbial community colonization and metabolic activity have significant effects on the healthy development of chicks (5). Unstable colonization of bacteria in chicks makes them more susceptible to bacterial infections (45), making the colonization of early beneficial bacteria very important. Previous studies have shown that dietary Bi^2^tos and inulin changed the number of intestinal bacteria, which was shown by a significant increase in the lactic acid bacterial count (37). Moreover, Mookiah et al. (46) revealed that dietary synbiotics can increase the counts of cecal *Lactobacillus* and *Bifidobacterium* and decrease *E. coli* abundance. However, there are few published reports on the response of intestinal bacteria to *in ovo* injection of synbiotics. Our results showed that the intra-amniotic synbiotic injection could increase *Lactobacillus* and *Bifidobacterium* colonization but decrease the population of *E. coli* in the chicken cecum. This beneficial effect may be attributed to early bacterial colonization facilitated by the *in ovo* injection of synbiotics. Hence, our findings showed that using *L. plantarum* and APS as synbiotics can promote the early colonization of beneficial bacteria in chicks and reduce pathogen entry. Our results of cecal *Lactobacillus, Bifidobacterium*, and *E. coli* provided new insights into the intra-amniotic effects of synbiotics in layer chicks. However, using molecular techniques in further research is expected to reveal more details regarding the cecal microflora.

## Conclusions

Our study showed that synbiotics had superior benefits compared to probiotics alone on chicks' growth performance, intestinal histomorphological parameters, and intestinal microflora population. The *in ovo* injection of synbiotics helped to maintain a beneficial intestinal microflora and augmented the host's immune system to provide a healthy gastrointestinal environment. Additionally, enhanced immune organ index and gut function resulted in improved weight gain and performance of the chicks without causing negative effects.

## Data Availability Statement

All data generated or analyzed during this study are available from the corresponding author on reasonable request.

## Ethics Statement

This study was conducted following recommendations of the Jilin Agriculture University Institutional Animal Care and Use Committee (JLAU08201409), and the experimental procedures were performed in compliance with the National Institutes of Health Guide for the Care and Use of Laboratory Animals (NIH Publications No. 8023).

## Author Contributions

S-bY and W-mL designed the study. Y-jQ, XM, and L-gX assisted with data analysis. A-yD and A-qJ performed animal tests, interpreted the results, and wrote the manuscript draft. All authors have read and approved the manuscript.

## Conflict of Interest

The authors declare that the research was conducted in the absence of any commercial or financial relationships that could be construed as a potential conflict of interest.

## Abbreviations

APS: Astragalus polysaccharide
BW: body weight
EW: initial egg weight
HW: hatching weight
FI: feed intake
BWG: body weight gain
FCR: the ratio of FI to BWG
IL-2: interleukin interleukin-2
IFN-γ: interferon-γ
SIgA: secretory immunoglobulin A
PBS: phosphate-buffered saline
BSA: bovine serum albumin
VH: villus height
CD: depth of crypt
HE: hematoxylin–eosin.

## References

[1] Pender CM Kim S Potter TD Ritzi MM Young M Dalloul RA. *In ovo* supplementation of probiotics and its effects on performance and immune-related gene expression in broiler chicks. Poultry Sci. (2017) 96:1052–62. 10.3382/ps/pew381 28158826

[2] Hou T Tako E. The *in ovo* feeding administration (Gallus Gallus)-an emerging *in vivo* approach to assess bioactive compounds with potential nutritional benefits. Nutrients. (2018) 10:418. 10.3390/nu10040418 29597266 PMC5946203

[3] Cardeal PC Caldas EOL Lara LJC Rocha JSR Baião NC Vaz DP. *In ovo* feeding and its effects on performance of newly-hatched chicks. World's Poultry Sci J. (2015) 71:655–62. 10.1017/S0043933915002445

[4] Siwek M Slawinska A Stadnicka K Bogucka J Dunislawska A Bednarczyk M. Prebiotics and synbiotics – *in ovo* delivery for improved lifespan condition in chicken. BMC Vet Res. (2018) 14:402. 10.1186/s12917-018-1738-z 30558599 PMC6296066

[5] Calik A Ergün A. Effect of lactulose supplementation on growth performance, intestinal histomorphology, cecal microbial population, and short-chain fatty acid composition of broiler chickens. Poultry Sci. (2015) 94:2173–82. 10.3382/ps/pev182 26188035

[6] Lan Y Verstegen MWA Tamminga S Williams BA. The role of the commensal gut microbial community in broiler chickens. World's Poultry Sci J. (2005) 61:95–104. 10.1079/WPS200445

[7] Cheled-Shoval SL Amit-Romach E Barbakov M Uni Z. The effect of *in ovo* administration of mannan oligosaccharide on small intestine development during the pre- and posthatch periods in chickens. Poultry Sci. (2011) 90:2301–10. 10.3382/ps.2011-01488 21934014

[8] Oliveira J Hoeven-Hangoor E Linde I Montijn RC Vossen JM. *In ovo* inoculation of chicken embryos with probiotic bacteria and its effect on posthatch Salmonella susceptibility. Poultry Sci. (2014) 93:818–29. 10.3382/ps.2013-03409 24706958

[9] Peebles ED. *In ovo* applications in poultry: a review. Poultry Sci. (2018) 97:2322–38. 10.3382/ps/pey081 29617899

[10] Stefaniak T Madej JP Graczyk S Siwek M Łukaszewicz E. Kowalczyk A. Selected prebiotics and synbiotics administered in ovo can modify innate immunity in chicken broilers. BMC Vet Res. (2019) 15:105. 10.1186/s12917-019-1850-8 30943971 PMC6448256

[11] Cox C Dalloul R. Immunomodulatory role of probiotics in poultry and potential *in ovo* application. Beneficial Microbes. (2015) 6:45–52. 10.3920/BM2014.0062 25213028

[12] Li Y Xu Q Yang C. Effects of probiotics on the growth performance and intestinal micro flora of broiler chickens. Pakistan J Pharmaceut Sci. (2014) 27:822–35. 10.1016/j.euroneuro.2013.09.011 24816710

[13] Pereira R Bortoluzzi C Durrer A Fagundes NS Pedroso AA Rafael JM. Performance and intestinal microbiota of chickens receiving probiotic in the feed and submitted to antibiotic therapy. J Animal Physiol Animal Nutrition. (2019) 103:72–86. 10.1111/jpn.13004 30485573

[14] Di Cerbo A Palmieri B Aponte M Morales-Medina JC Iannitti T. Mechanisms and therapeutic effectiveness of lactobacilli. J Clin Pathol. (2016) 69:187–203. 10.1136/jclinpath-2015-202976 26578541 PMC4789713

[15] Brisbin JT Gong J Parvizi P Sharif S. Effects of lactobacilli on cytokine expression by chicken spleen and cecal tonsil cells. Clin Vaccine Immunol. (2010) 17:1337–43. 10.1128/CVI.00143-10 20668142 PMC2944448

[16] Noujaim JC Andreatti Filho RL Lima ET Okamoto AS Amorim RL Torres Neto R. Detection of T lymphocytes in intestine of broiler chicks treated with lactobacillus spp. and challenged with Salmonella enterica Serovar Enteritidis. Poultry Sci. (2008) 87:927–33. 10.3382/ps.2007-00476 18420983

[17] Zheng Y Ren W Zhang L Zhang Y Liu D Liu Y. A review of the pharmacological action of astragalus polysaccharide. Front Pharmacol. (2020) 11:349. 10.3389/fphar.2020.00349 32265719 PMC7105737

[18] Xie JH Jin ML Morris GA Zha Q Chen HQ Yi Y. Advances on bioactive polysaccharides from medicinal plants. Critical Rev Food Sci Nutrition. (2016) 56:S60–84. 10.1080/10408398.2015.1069255 26463231

[19] Shan C Sun B Dalloul RA Zhai Z Sun P Li M. Effect of the oral administration of astragalus polysaccharides on jejunum mucosal immunity in chickens vaccinated against Newcastle disease. Microbial Pathogenesis. (2019) 135:103621. 10.1016/j.micpath.2019.103621 31310831

[20] Li SP Zhao XJ Wang JY. Synergy of Astragalus polysaccharides and probiotics (Lactobacillus and Bacillus cereus) on immunity and intestinal microbiota in chicks. Poultry Sci. (2009) 88:519–25. 10.3382/ps.2008-00365 19211520

[21] Samanta AK Senani S Kolte AP Sridhar M Jayapal N. Effect of Prebiotic on digestibility of total mixed ration. Indian Vet J. (2012) 89:41–2. Available online at: https://www.researchgate.net/publication/288997449_Effect_of_Prebiotic_on_digestibility_of_total_mixed_ration

[22] Dai D Wu S Zhang H Qi G Wang J. Dynamic alterations in early intestinal development, microbiota and metabolome induced by in ovo feeding of L-arginine in a layer chick model. J Anim Sci Biotechnol. (2020) 11:19. 10.1186/s40104-020-0427-5 32175081 PMC7063725

[23] Dunislawska A Slawinska A Bednarczyk M Siwek M. Transcriptome modulation by in ovo delivered Lactobacillus synbiotics in a range of chicken tissues. Gene. (2019) 698:27–33. 10.1016/j.gene.2019.02.068 30831211

[24] Tian G Zhao M Zhang L Li J Yu L Gao F.. *In ovo* feeding of L-arginine regulates intestinal barrier functions of posthatch broilers by activating the mtor signaling pathway. J Sci Food Agriculture. (2018) 98:1416–25. 10.1002/jsfa.8609 28771730

[25] Sobolewska A Bogucka J Dankowiakowska A Elminowska-Wenda G Stadnicka K Bednarczyk M. The impact of synbiotic administration through in ovo technology on the microstructure of a broiler chicken small intestine tissue on the 1 st and 42 nd day of rearing. J Anim Sci Biotechnol. (2017) 8:61. 10.1186/s40104-017-0193-1 28781771 PMC5537990

[26] Cleusix V Lacroix C Dasen G Leo M Le Blay. G. Comparative study of a new quantitative real-time PCR targeting the xylulose-5-phosphate/fructose-6-phosphate phosphoketolase bifidobacterial gene (xfp) in faecal samples with two fluorescence in situ hybridization methods. J Appl Microbiol. (2010) 108:181–93. 10.1111/j.1365-2672.2009.04408.x 19566721

[27] Heilig HG Zoetendal EG Vaughan EE Marteau P Akkermans ADL. Molecular diversity of lactobacillus spp. and other lactic acid bacteria in the human intestine as determined by specific amplification of 16S ribosomal DNApplied A. Environ Microbiol. (2002) 68:114–23. 10.1128/aem.68.1.114-123.2002 11772617 PMC126540

[28] Walter J Tannock GW Tilsala-Timisjarvi A Rodtong S Loach DM Munro K. Detection and identification of gastrointestinal lactobacillus species by using denaturing gradient gel electrophoresis and species-specific PCR primers. Appl Environ Microbiol. (2000) 66:297–303. 10.1128/aem.66.1.297-303.2000 10618239 PMC91821

[29] Pham VH Kan L Huang J Geng Y Zhen W Guo Y. Dietary encapsulated essential oils and organic acids mixture improves gut health in broiler chickens challenged with necrotic enteritis. J Anim Sci Biotech. (2020) 11:1–18. 10.1186/s40104-019-0421-y 32110391 PMC7033934

[30] Sun H Ni X Song X Wen B Zhou Y Zou F.. Fermented yupingfeng polysaccharides enhance immunity by improving the foregut microflora and intestinal barrier in weaning rex rabbits. Appl Microbiol Biotech. (2016) 100:8105–20. 10.1007/s00253-016-7619-0 27260288

[31] Yang CM Cao GT Ferket PR Liu TT Zhou L Zhang L. Effects of probiotic, *Clostridium butyricum*, on growth performance, immune function, and cecal microflora in broiler chickens. Poultry Sci. (2012) 91:2121–9. 10.3382/ps.2011-02131 22912445

[32] Higgins SE Higgins JP Wolfenden AD Henderson SN Torres-Rodriguez A Tellez G. Evaluation of a lactobacillus-based probiotic culture for the reduction of salmonella enteritidis in neonatal broiler chicks. Poultry Sci. (2008) 87:27–31. 10.3382/ps.2007-00210 18079446

[33] Flint JF Garner MR. Feeding beneficial bacteria: a natural solution for increasing efficiency and decreasing pathogens in animal agriculture1 1Papers from the Current and Future Prospects for Natural and Organic Poultry Symposium were presented at the Poultry Science Association's 97th Annual Meeting in Niagara Falls, Ontario, Canada. J Appl Poultry Res. (2009) 18:367–78. 10.3382/japr.2008-00133

[34] Teague KD Graham LE Dunn JR Cheng HH Anthony N Latorre JD. *In ovo* evaluation of FloraMax®-B11 on Marek's disease HVT vaccine protective efficacy, hatchability, microbiota composition, morphometric analysis, and Salmonella enteritidis infection in broiler chickens. Poultry Sci. (2017) 96:2074–82. 10.3382/ps/pew494 28160004

[35] Wang J Wang B Du H Zhang H Zhao X. Effects of diutina rugosa sd-17 on growth performance, intestine morphology, and immune status of chickens. Poultry Sci. (2019) 98:6311–8. 10.3382/ps/pez428 31350991 PMC8913761

[36] Ghahri H Toloei T Soleimani B. Efficacy of antibiotic, probiotic, prebiotic and synbiotic on growth performance, organ weights, intestinal histomorphology and immune response in broiler chickens. Global J Animal Sci Res. (2013) 1:25–41. Available online at: https://www.mendeley.com/catalogue/50101af8-607e-37a3-87a5-f792fb1c7730/

[37] Miśta D Króliczewska B Pecka-Kiełb E Kapuśniak V Zawadzki W Graczyk S. Effect of *in ovo* injected prebiotics and synbiotics on the caecal fermentation and intestinal morphology of broiler chickens. Anim Product Sci. (2017) 57:1884–92. 10.1071/AN16257

[38] Zhang ZF Kim IH. Effects of multistrain probiotics on growth performance, apparent ileal nutrient digestibility, blood characteristics, cecal microbial shedding, and excreta odor contents in broilers. Poultry Sci. (2014) 93:364–70. 10.3382/ps.2013-03314 24570458

[39] Farnell MB Donoghue AM de los Santos FS Blore PJ Hargis BM Tellez G. Upregulation of oxidative burst and degranulation in chicken heterophils stimulated with probiotic bacteria. Poultry Sci. (2006) 85:1900–6. 10.1093/ps/85.11.1900 17032821

[40] Lee K Lee S Lillehoj H Li G Jang S Babu U. Effects of direct-fed microbials on growth performance, gut morphometry, and immune characteristics in broiler chickens. Poultry Sci. (2010) 89:203–16. 10.3382/ps.2009-00418 20075271

[41] Rothkötter HJ Hriesik C Barman NN Pabst R. B and also T lymphocytes migrate via gut lymph to all lymphoid organs and the gut wall, but only IgA^+^ cells accumulate in the lamina propria of the intestinal mucosa. Eur J Immunol. (1999) 29:327–33. 10.1002/(SICI)1521-4141(199901)29:01<327::AID-IMMU327>3.0.CO;2-K 9933115

[42] Chee SH Iji PA Choct M Mikkelsen LL Kocher A. Functional interactions of manno-oligosaccharides with dietary threonine in chicken gastrointestinal tract. I. Growth performance and mucin dynamics. Br Poultry Sci. (2010) 51:658–66. 10.1080/00071668.2010.517251 21058070

[43] Luerce TD Gomes-Santos AC Rocha CS Moreira TG Cruz DN Lemos L. Anti-inflammatory effects of Lactococcus lactis NCDO 2118 during the remission period of chemically induced colitis. Gut Pathogens. (2014) 6:33. 10.1186/1757-4749-6-33 25110521 PMC4126083

[44] Sławińska A Siwek M Bednarczyk M. *In vitro* screening of immunomodulatory properties of synbiotics in chicken DT40 cell line. Anim Sci Papers Rep. (2016) 34:81–93. Available online at: https://www.researchgate.net/publication/298714037_In_vitro_screening_of_immunomodulatory_properties_of_synbiotics_in_chicken_DT40_cell_line

[45] Gaggìa F Mattarelli P Biavati B. Probiotics and prebiotics in animal feeding for safe food production. Int J Food Microbiol. (2010) 141:S15–28. 10.1016/j.ijfoodmicro.2010.02.031 20382438

[46] Mookiah S Sieo CC Ramasamy K Abdullah N Ho YW. Effects of dietary prebiotics, probiotic and synbiotics on performance, caecal bacterial populations and caecal fermentation concentrations of broiler chickens. J Sci Food Agriculture. (2014) 94:341–8. 10.1002/jsfa.6365 24037967

